# Detection of Polystyrene Microplastics up to the Single Nanoparticle Limit Using SERS and Advanced ANN Design (KANformer)

**DOI:** 10.1021/acssensors.5c00846

**Published:** 2025-06-25

**Authors:** Karolina Kukralova, Andrii Trelin, Elena Miliutina, Vasilii Burtsev, Vaclav Svorcik, Oleksiy Lyutakov

**Affiliations:** Department of Solid State Engineering, 52735 University of Chemistry and Technology, Prague 16628, Czech Republic

**Keywords:** PS microplastic, porous plasmon substrate, annealing, SERS, artificial neural network, KAN

## Abstract

Due to uncontrolled release, gradual accumulation, low degradation rate, and potential negative impact on human health, microplastics (MPs) pose a serious environmental and healthcare risk. Thus, the spread of MPs should be at least carefully monitored to identify and eliminate their main sources, as well as to provide a suitable alarm in the case of MP concentration increase. Among various detection methods, surface-enhanced Raman spectroscopy (SERS) poses a unique detection limit and the ability to perform outdoor measurements without preliminary sample treatment. However, the utilization of SERS for MPs detection is significantly limited for a few reasons. First, the maximal SERS enhancement occurs in the so-called hot spots, where the MPs cannot penetrate due to their size. In addition, the natural environment can produce a significant spectral background, which blocks the microplastic characteristic signal. To overcome these limitations, we propose a new alternative route for introduction of MPs into the plasmonic hot spots, using in situ MP annealing and an advanced artificial neural network (ANN) design, the Kolmogorov–Arnold transformer (KANformer, KANF). Polystyrene (PS) MPs were used as a model compound. We have also demonstrated the potential versatility of our approach using different microplastics, such as polyethylene, polypropylene, and polyethylene terephthalate. The proposed approach allows us to detect the presence of PS up to the single nanoparticle limit (in the mL of analyzed solution) with a probability of above 95%, even under mixing with groundwater model matrices.

## Graphical abstract

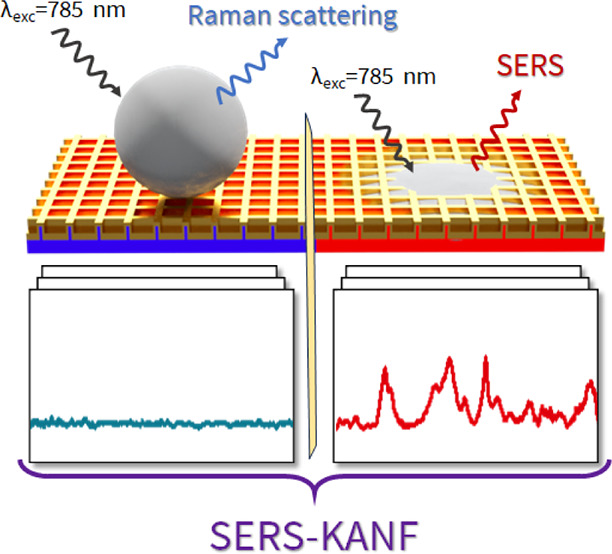

As a result of the increased use of polymeric materials, the concentration of microplastics (MPs) in the environment has increased significantly in recent years and has reached alarming levels. The presence of MPs has been identified in a variety of environmental matrices, including air, drinking water, seawater, soil, and food.^1–8^ Because of their relatively small size and low biodegradability, MPs can accumulate in the food chain and cause several diseases in both animals and humans.^9–12^ Therefore, the concentration of MPs should be carefully monitored in various media, ideally using simple, reliable approaches. However, currently, there are no standardized methods for the detection of MPs in water.^13,14^ Therefore, the development of reliable identification and quantification methods for MPs in the environment is urgently required.

The current methods for the detection of MP presence and determination of their chemical composition encompass a range of techniques, including optical and electron microscopies.^15,16^ Indeed, visual inspection methods can provide information about the physical properties of MPs, such as their size and shape, but cannot reveal their chemical composition.^17,18^ To overcome this limitation, pyrolysis-gas chromatography–mass spectrometry (Py-GC-MS) or liquid chromatography–tandem mass spectrometry (LC-MS/MS) can be used.^19–22^ These methods offer high detection reproducibility and sensitivity, but they are limited by expensive equipment, complicated sample pretreatment, and skilled analytical processes, making it impossible to perform express outdoor detection.^23^ Thermal MP analysis coupled with mass spectrometry and a variety of vibrational spectroscopic techniques has also been employed.^24,25^ Because of their simplicity and availability, vibrational spectroscopies such as FTIR or Raman spectroscopy are the most employed methods for the detection of MPs in water.^14,16^ However, for utilization of spectroscopic methods, the samples need to be purified and dried before measurement.^26,27^ Additionally, the sensitivity and detection limits of FTIR and Raman spectroscopies are far from adequate.^28,29^ In the case of Raman spectroscopy, lower sensitivity can be significantly enhanced with utilization of surface-enhanced Raman spectroscopy (SERS), which was also proposed for MP detection.^30^

Recent research using SERS-based MP detection mainly focuses on the sensitive quantification of MP concentration, detection of various MP sizes, and classification of the MP composition in the sample matrix.^31–33^ In a common experimental approach, plasmon-active nanoparticles are attached to the surface of MPs, and the detection wavelength is adjusted to efficiently excite the plasmon resonance.^30,34–37^ However, considering the typical range of the plasmonic evanescent wave, in this case, the spectral information can be read from a distance of up to several nanometers and can rather correspond to the molecules adsorbed on the MP surface, as shown in Scheme 1A.^38–40^ As a result, the SERS will rather reveal the composition of the MP surface layer, and taking into account the ability of MPs to adsorb various molecules, this analysis may not provide the real information about the presence of the MPs or their composition. Similar problems can be expected in the case of simple deposition of MPs onto more sophisticated SERS substrates, composed of patterned surfaces with locally excited hot spots providing information from the 2–5 nm layer.^41–44^

**Scheme 1:**
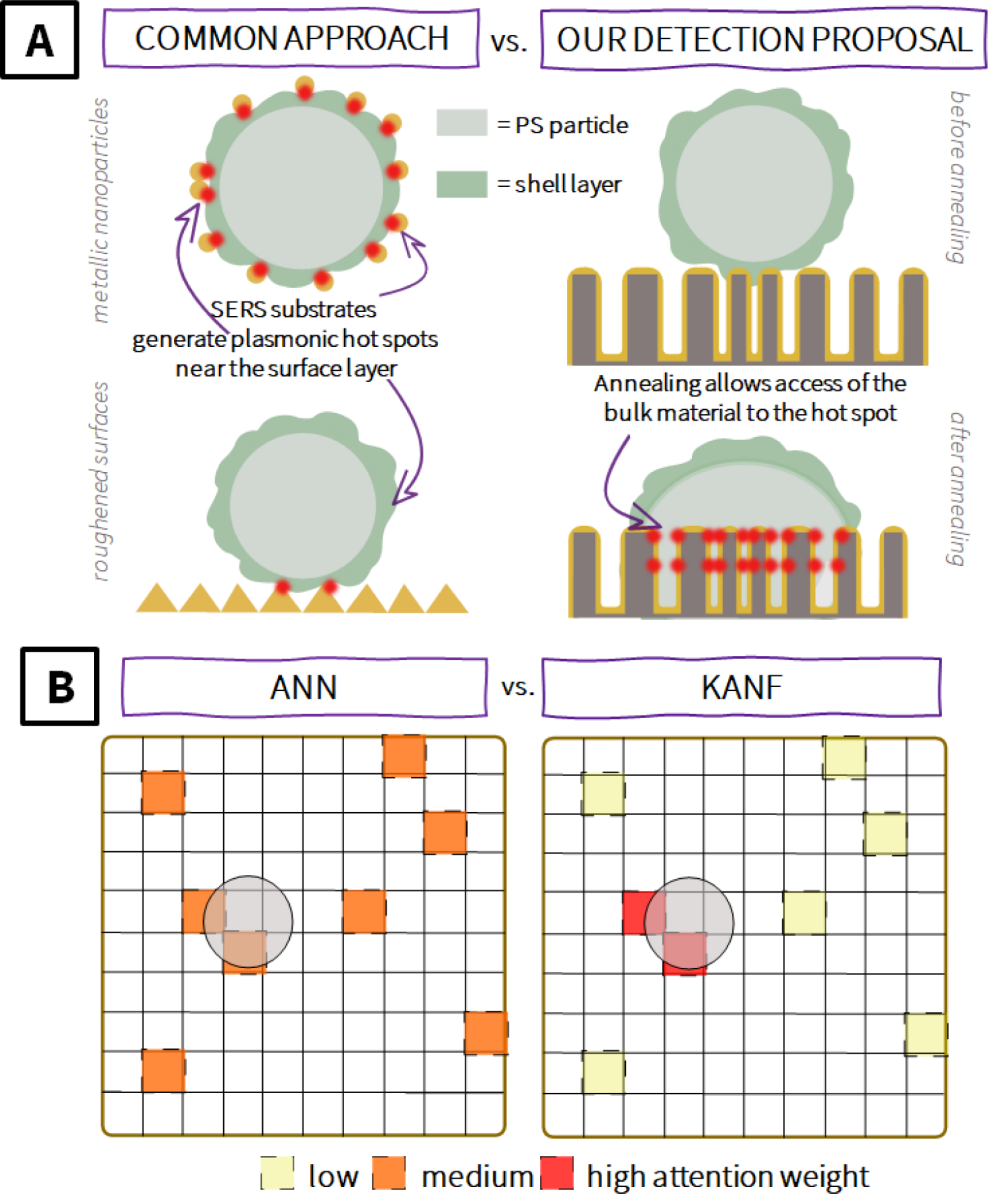
Representation of the Advantages of Our Work^a^

a (a) Common detection vs. detection proposed in this work. In a common case, the information is read from plastics surface and may not correspond to the “Real” composition of the plastic. In contrast, in our detection proposal, the information is read from the bulk material, since PS materials penetrate into plasmon-active pores. (b) Comparison of how many spectra are considered and how many of them carry information about MP presence: common ANN (8 from 100 spectra, all spectra are equally weighted) vs. KANF (8 from 100, attention weighed). The weight of the given spectrum is represented by its colour.

To overcome this drawback and to tune SERS detection toward more practical applications, several approaches were considered. One of them is based on creation of a surface with a specific shape, for example, with some cavities that can trap the MPs.^45–47^ In this case, the analytical signal is produced by “bulk” MP materials.

As an alternative, the deposited MPs can be transferred into organic solvents, where their dissolution or swelling enables better contact with the plasmon-active SERS substrate.^41^ Additionally, a pretreatment of the shell layer of MPs can be used, meeting two goals simultaneously – to reveal the bulk material and provide sites for plasmon-active nanoparticle attachment.^33,48,49^ These approaches allow plasmonic hot spots to be filled with the polymer material. However, they cannot be considered universal, since it is impossible to select a universal organic solvent or treatment agent for all the plastics due to the differences in plastics’ polarity and the potential presence of three-dimensional cross-linked networks in particles.

In general, SERS meets the main requirements for portable, simple, and fast outdoor detection, especially considering the significant progress in the preparation of a simple and scalable SERS substrate.^50–52^ However, taking into account the surface sorption of MPs, the information read from SERS can lead to an incorrect conclusion.^53–55^ In this work, we propose a simple solution aimed at overcoming this drawback, i.e., gradual heating of deposited MPs, which undergo glass transition, and the softened material fills plasmon-active voids. Hence, the signal from the bulk of MPs can be obtained in a simple and scalable way (see Scheme 1). On the other hand, the other molecules present in the real samples will also penetrate into the SERS substrate’s pores and produce significant background. Thus, we additionally propose the utilization of an artificial neutral network (ANN) design for evaluation of the SERS spectra.^56–62,69–72^

It should be taken into account that the main challenge in SERS detection of MPs is their insufficient interaction with plasmonic hot spots, so that only a small portion of collected spectra contains useful information about the presence of MPs (as depicted in Scheme 1B). To address this issue, the NN architectures (in particular, attention-based models, transformers) which allow simultaneous processing of multiple spectra, such as transformer-based models can be used.^60,67,73^ Transformer-like ANNs operate on an arbitrary number of input spectra, combine information from all collected spectra, and provide a conclusion on the examined sample as a whole. By analyzing multiple spectra simultaneously, the accuracy of SERS-KANF detection is enhanced significantly, and advantages of the SERS and ANN techniques are fully utilized. In particular, KANF allows us to consider particular SERS spectra in an “attention weighted” regime, i.e., it makes decisions based on more important spectra, i.e., those containing information on the PS presence (spectra with “high attention weight“, Scheme 1B) and partially ignoring the useless spectra (spectra with low attention weight).

## Experimental Section

Detailed descriptions of the materials used and the characterization techniques are given in Supporting Information.

### SERS Substrate Preparation

The SERS substrates were prepared by a previously described method.^74^ Briefly, the p-type silicon (100) wafer with low resistivity was cleaned and electrochemically etched in an HF:DMF solution (ratio 1:20) under the following conditions: 2 mA, 15 min. The etched wafer was rinsed with ethanol and dried. As the next step, the wafer was coated with a Au layer using metal sputtering (direct current; Ar plasma; gas purity: 99.995%; pressure: 4 Pa; discharge power: 7.5 W; sputtering time: 300 s; current: 40 mA).

### Sample Preparation

Polystyrene was used in the form of microspheres (PS, latex, 2.5 wt % dispersion in water, diameter 0.5 μm, Alfa Aesar). Alternatively, 200 nm and 5 μm PS nanoparticles from Thermo Fisher and Magsphere were used for control experiments.

The LDPE, PP, and PET plastics were obtained from GoodFellow. To decrease the size of polymer particles (i.e., to create microplastics), the materials were subjected to mechanical grinding.

The SERS substrates were cut into 4 × 4 mm^2^ samples, onto which 10 μL of a solution containing PS (from 10^4^ to 10^8^ particles/L) was drop-deposited. When stated, the solution also contained background substances (HA, AA, TA, or their mixture at concentrations of 30 or 10 mg/L). The samples were heated to an elevated temperature (from 50 to 150 °C) to allow the polymer to penetrate the pores of the SERS substrate and to take advantage of the plasmonic enhancement of the signal from the polymer. At the end, the samples were cooled and subjected to SERS measurements.

To prepare the real samples, water from outdoor sources was used (in two different places in Prague – Hloubětín and Dejvice – and one in a protected nature area in Brdy). The water was filtered through a 10 nm filter to remove potential microplastic nanoparticles. After that, a microplastic suspension (PS, 500 nm) was added to obtain the final concentrations of 10^5^, 10^6^, and 5 × 10^7^. Some samples were left without PS addition. In the next step, the samples were processed and measured in a similar way to the model samples, with the addition of humic organic acids.

## Results and Discussion

In this study, we propose the use of plasmon-active substrates and surface-enhanced Raman spectroscopy in combination with a novel neural network – Turbo Transformer KAN (KANformer or KANF for brevity) – for the detection of MPs in water, including simulated groundwater. A schematic representation of the proposed experimental concept is given in Figure 1. First, the porous silicon (pSi) surface was created by electrochemical etching (Figure S1). To introduce plasmon activity and enable SERS detection in pSi, the surface was covered with a thin layer of Au (pSi@Au) using the previously optimized approach.^74^ As a result, the SERS enhancement factor obtained on these substrates reached a value ≈ 10^6^ (Figure S2). In the next step, the PS microparticles (500 nm in size – Figure S3) were mixed with water or simulated groundwater and drop-deposited on the SERS substrate. However, subsequent SERS measurements (Figure S4) indicated no difference between PS particles deposited on a flat Si surface or on the plasmon-active surface. In this case, no SERS enhancement occurred. An even worse situation was observed for a low concentration of PS particles, where the PS response was screened by the SERS signal from the “background” molecules (Figure S5 and related remarks in the Supporting Information). We assumed that the PS microparticles did not fill the pores in the plasmon-active substrate, and thus, SERS enhancement did not occur. To obtain a better response, annealing of the samples was proposed before SERS measurements, with the aim of filling the plasmon-active pores with the melted PS material (Figure 1A). After optimization of the annealing procedure and determination of the optimal temperature and time, the SERS database was collected (Figure 1B) and used for training and validation of the KANF (Figure 1C). The composition of the samples and the number of SERS spectra measured for KANF are summarized in Table 1. Finally, the results of the SERS-KANF approach were checked using blinded validation, where samples with unknown PS concentrations were measured by SERS and then subjected to KANF analysis.

**Figure 1:**
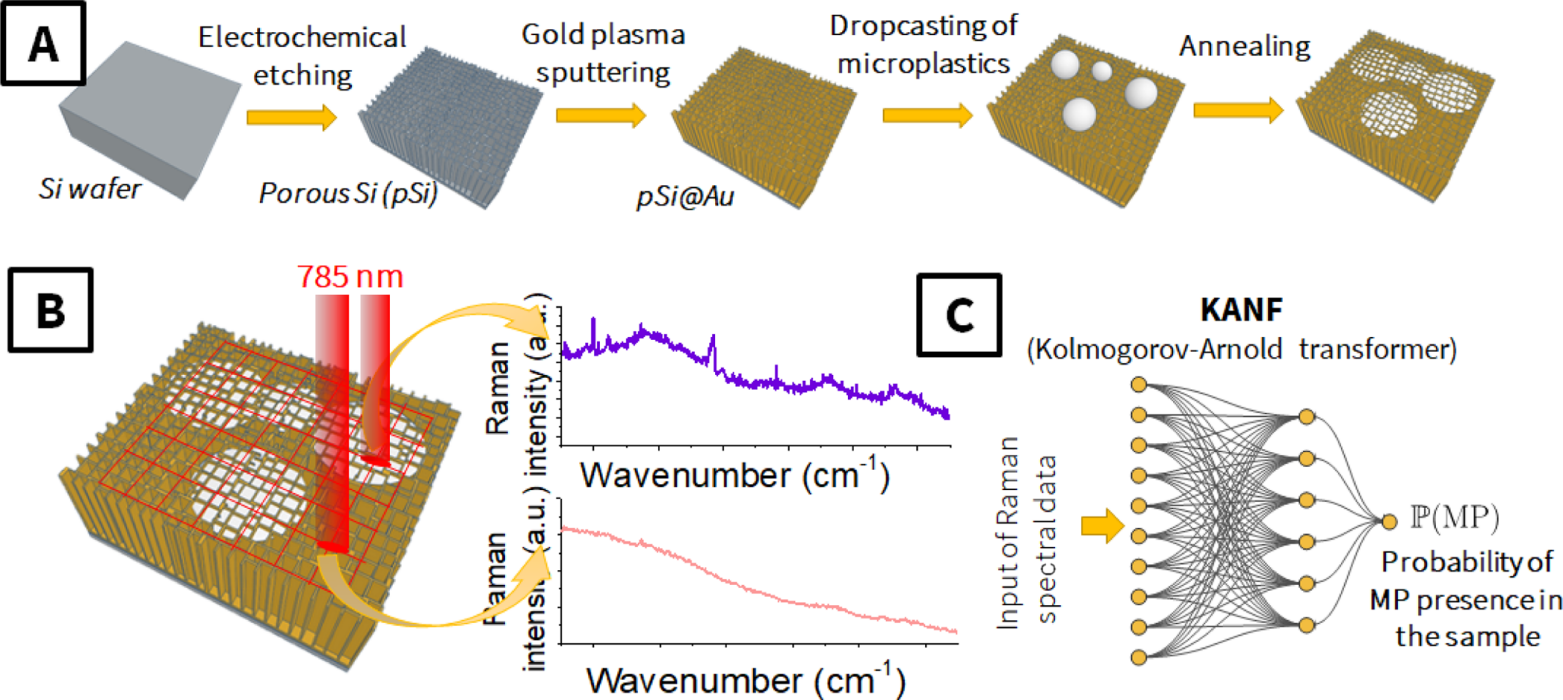
A schematic representation of the experimental concept: (A) particular steps of sample preparation; (B) mapping the sample surface and collecting Raman spectra; (C) data processing scheme in which the spectra are evaluated by KANF and the probability of MP presence in the sample is calculated.

**Table 1:**
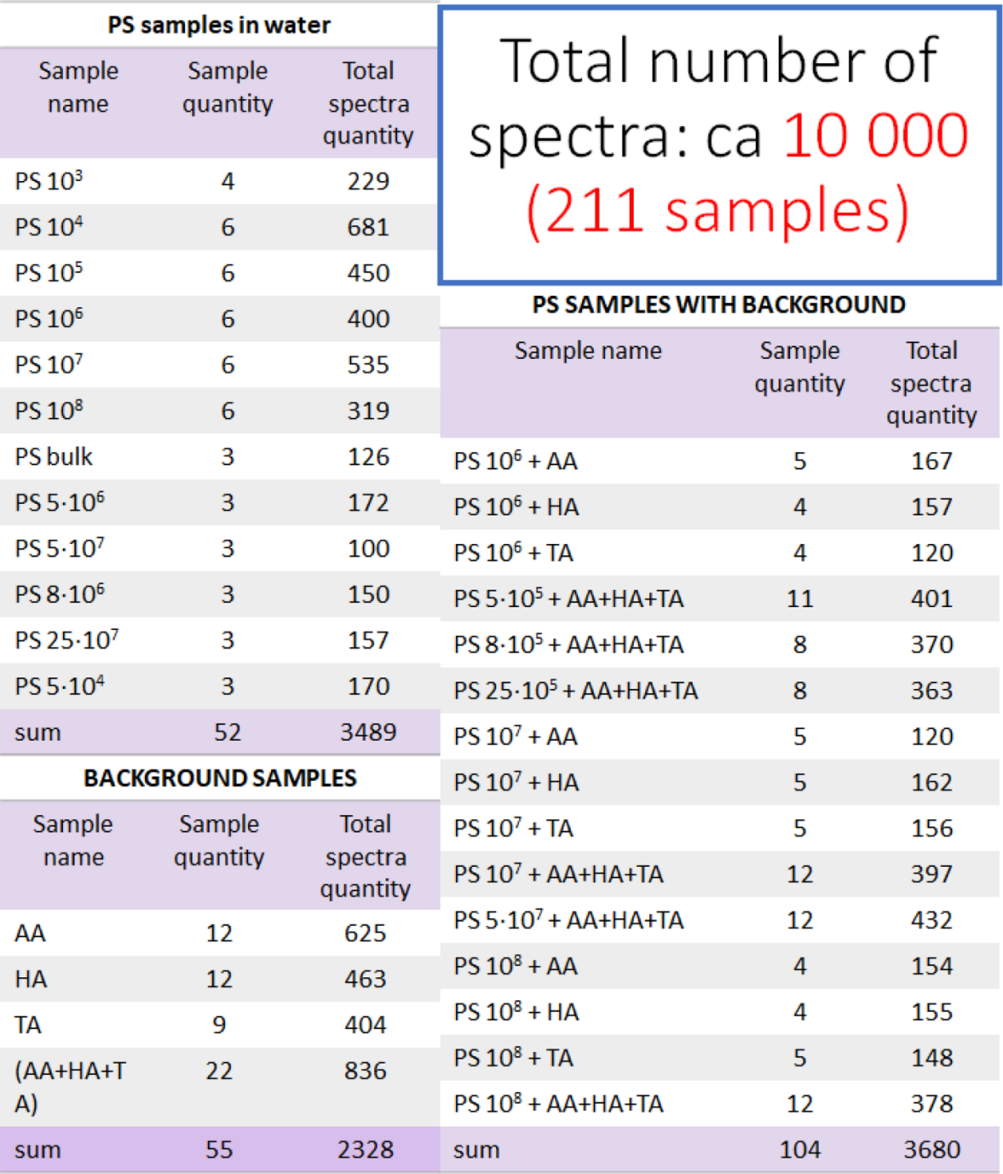
Composition of Samples and Amounts of SERS Spectra Used for KANF Training and Validation

The characterization of the SERS substrates is presented in Figure 2. First, the SEM image (Figure 2A) indicates the porous structure of the surface, which can support the excitation of local plasmons within the pores. The pore lateral size is in the 50–110 nm range, so PS microparticles, 500 nm in diameter (Figure S3), cannot directly penetrate the pores. The excitation of localized plasmon(s) was confirmed by UV–vis measurements performed in the back-reflected light. As is evident from Figure 2B, the deposition of the Au layer on the etched Si surface results in the appearance of a wide absorption band located near 650 nm. This band should be related to local plasmon excitation. In addition, the quasi-ordered surface pattern (evident from SEM) will ensure the homogeneous distribution of plasmonic hot spots, thereby ensuring the homogeneity of the SERS response. This assumption was additionally checked with the utilization of the model SERS analyte, crystal violet (CV), deposited on the pSi@Au by spin coating. Mapping of CV across 2 × 2 mm^2^ resulted in the production of a relatively convergent SERS response (Figure 2C)—the deviation of the characteristic peak intensity did not exceed 17%. In turn, the calculated SERS EF was found to be 10^6^ (see Supporting Information for detailed information). Surface wettability measurements were also performed using water and glycerol drops. Surface interaction with different polar liquids is expected to affect the penetration of nonpolar PS materials inside the pores as well. The contact angles on pSi@Au were found to be 78.9 ± 1.5° for water and 39.6 ± 4.9° for glycerol, indicating the slightly hydrophobic nature of the surface, which can support the penetration of nonpolar PS inside the pores of pSi@Au.

**Figure 2:**
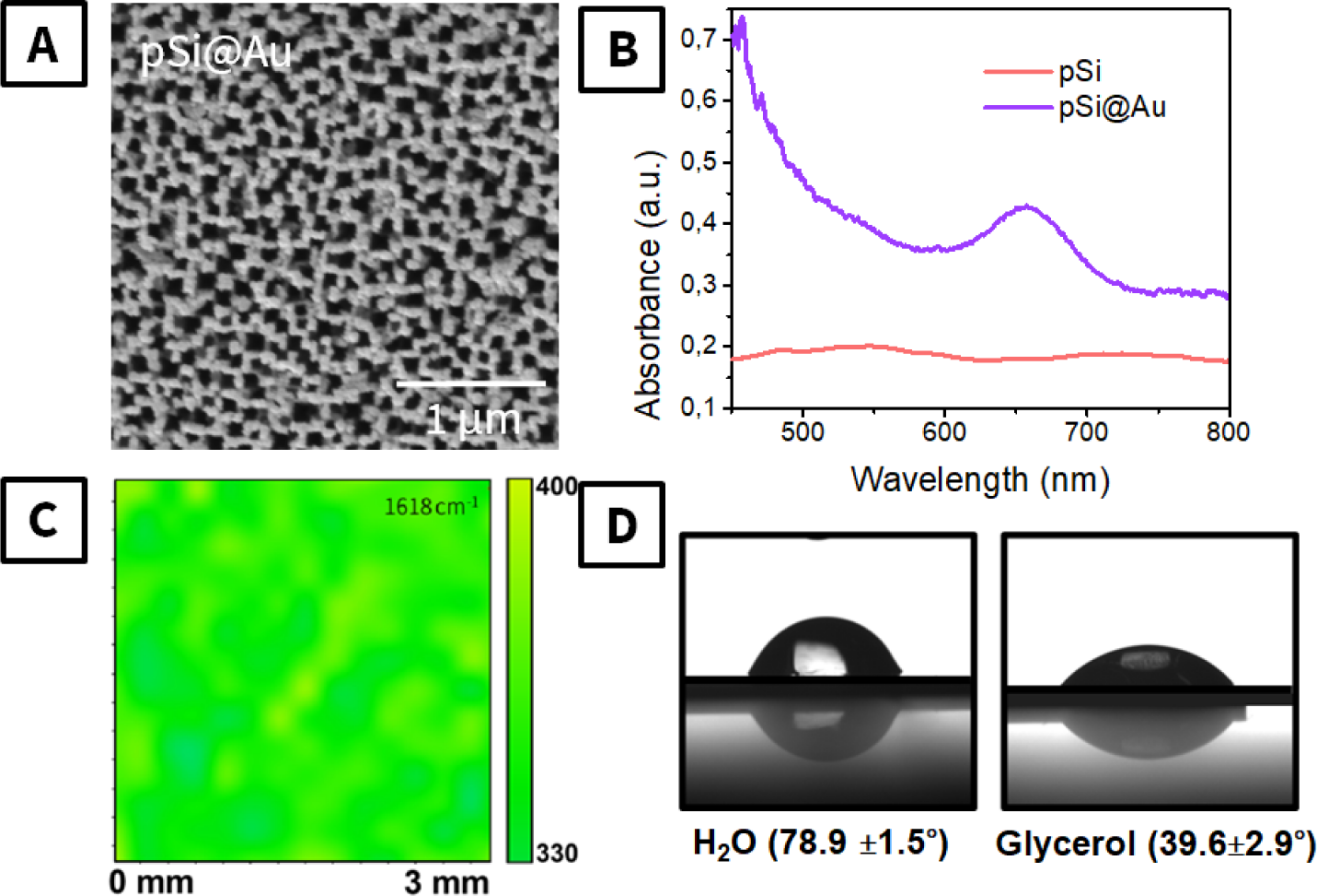
Characterization of the SERS substrates: (A) SEM image of pSi coated with Au; (B) UV–vis spectra of pSi before and after the Au deposition; (C) SERS mapping of CV response over the sample area, map of peak at 1618 cm^–1^; (D) wettability tests performed on pSi@Au with utilization of water and glycerol and corresponding contact angles.

In the next step, we investigated the behavior of PS microspheres on the porous SERS-active substrate. First, we determined the glass transition of PS using differential scanning calorimetry (Figure 3A). The obtained results indicate that PS undergoes glass transition in the 100–120 °C temperature range, evident as an apparent peak of heat consumption. At this temperature, the softening of PS particles can be expected, which will be accompanied by the deformation of the particle shape and subsequent pSi@Au pore filling. This behavior of PS particles was confirmed by SEM images of PS particles on the pSi@Au, where the transition of the initially spherical PS particles to flat islands is evident (Figure 3B). Such deformation of PS microparticles can be associated with the filling of the porous structure by the softened polymer. In situ SERS measurements as a function of temperature are presented in Figure 3C,D. As expected, the spectra from as-deposited microparticles (without heating) showed no characteristic peaks, since the PS cannot be triggered by local plasmon and thus cannot produce a SERS signal. A similar situation was observed up to annealing at 80 °C, which is the temperature range “below” the glass transition temperature. At 90 °C, we observed the appearance of characteristic PS peaks, and their intensity increased with annealing temperature up to 120 °C. Further temperature increases resulted in only a slight decrease of the characteristic peak intensity, probably due to partial polymer degradation. So, taking into account the results presented in Figure 3, we can suppose that the glass transition of PS leads to its softening and gradual filling of plasmon-active pores, spatial overlapping of PS and plasmonic hot spots, and, in turn, the appearance of a SERS signal.

**Figure 3:**
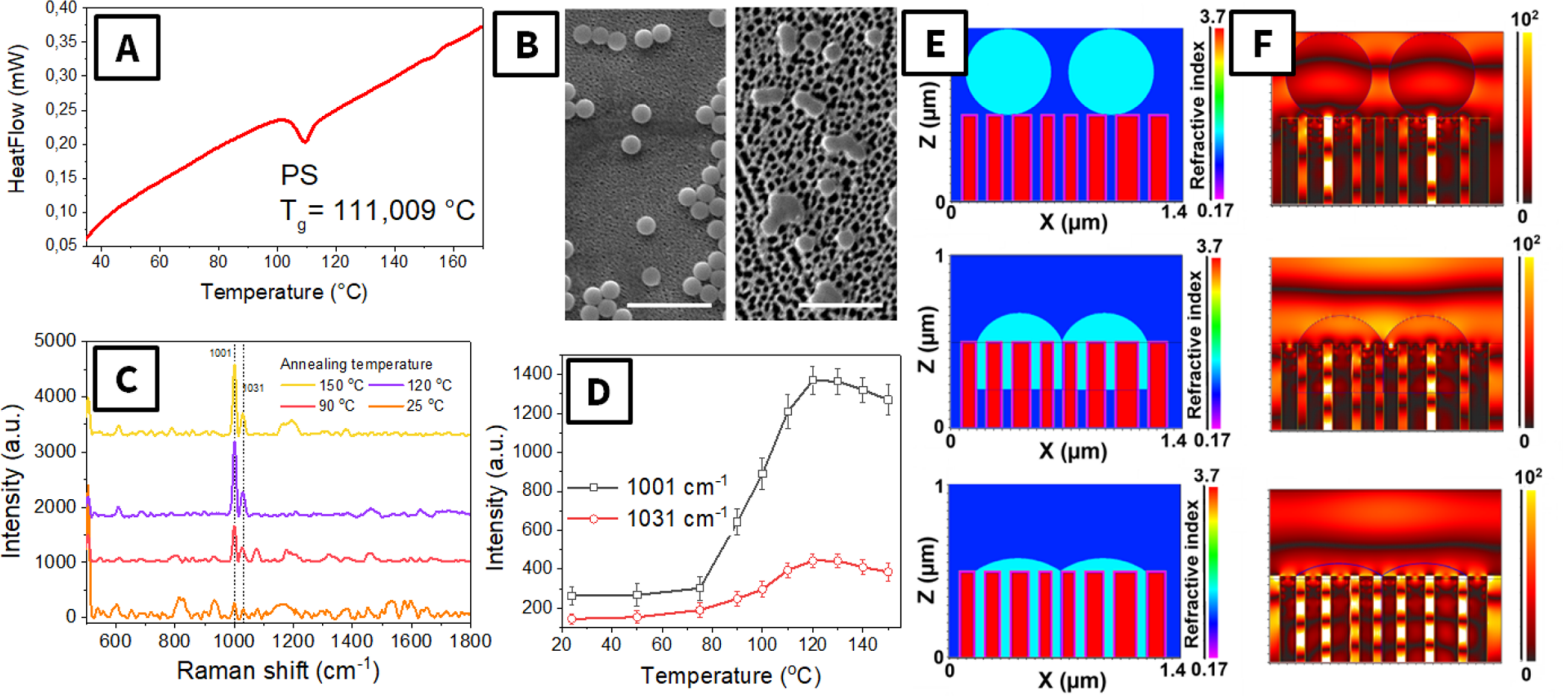
(A) DSC curves of PS microparticles; (B) SEM images of the PS microparticle before (left) and after (right) the annealing at 120 °C (scale bar: 2.5 μm); (C) averaged SERS spectra as a function of annealing temperature; (D) dependence of the characteristic intensity of the PS SERS peak (1001 and 1031 cm^–1^) on the annealing temperature; (E, F) material structure (gradual filling of pSi@Au pores with PS) used for TDFD simulation and obtained distribution of the plasmon-related electric field.

We also tested the annealing time required for reaching the maximal SERS response. In particular, the results of time-resolved experiments for different PS nanoparticle sizes are presented in Figure S6. As is evident, the use of annealing leads to a gradual increase in the SERS signal intensity, which correlates with the filling of the plasmon-active pores. After a certain amount of time, the peak intensity reaches a plateau and does not increase anymore. This phenomenon can occur for two reasons: all plasmon-active pores are filled (in the case of excess polymer material) or all the material is already in the pores (in the case of fewer PS nanoparticles). The typical time required to reach the SERS plateau is in the range of 20–30 min, which is not surprising, since polymers are quite viscous materials. In turn, the saturation time is a function of the nanoparticle size used (this can be associated with both the molecular weight of the material and its thermodynamic stability, determined by the surface-to-volume ratio). In turn, annealing at a higher temperature (for a longer time) results in the decrease of SERS intensity. This effect can be associated with the gradual degradation of the material (annealing was performed in air) and the loss of its mass (the results of the control gravimetric tests are shown in Figure S7), both leading to a lower SERS intensity.

For further confirmation, we performed a range of numerical simulations (Figure 3E,F) to prove the overlap of plasmon-related evanescent waves and PS gradually penetrating into the pores. The simulation of the pristine plasmon-active substrate (with deposited PS spherical particles) indicates that the main plasmon energy is concentrated inside the pores. In this case, the interaction of plasmonic hot spots with the as-deposited PS particles is minimal (Figure 3E,F, top line), a finding that corresponds well with the absence of a SERS signal. However, when the pSi@Au pores are filled with PS after annealing, efficient overlap of the plasmon wave and PS materials occurs, which results in the production of an intensive SERS signal (also correlating well with temperature-dependent SERS measurements).

To further demonstrate the ability to introduce the MP material inside the plasmonic hot spots, we performed additional experiments with the utilization of PS particles of different sizes and several alternative types of MPs. First, the results of PS with 5 μm and 200 nm were analyzed using a similar approach, i.e., sample heating and subsequent SERS analysis (Figure S8). The obtained results were similar to the previously observed ones – close to zero signal without annealing and a gradual increase of the characteristic band intensity with time under annealing at 120 °C.

In turn, results of experiments performed with utilization of low-density polyethylene (LDPE), polypropylene (PP), and polyethylene terephthalate (PET) microplastics are presented in Figure 4. In this case, a gradual increase of the characteristic SERS peak intensity with the increase of annealing time was also observed. Comparison of the obtained results with DSC curves indicates good agreement between the glass transition temperature and the peak intensity increase. So, the proposed approach can be used even in the case of alternative MPs, where the MP heating directly on a mesoporous SERS substrate allows to fill plasmonic hot spots and to detect their presence. In other words, the proposed approach is universal and can be used for a whole range of plastics without the need for preliminary processing. In the next step, we mainly focused on the utilization of the SERS-ANN approach for PS (to keep the article to a reasonable length).

**Figure 4:**
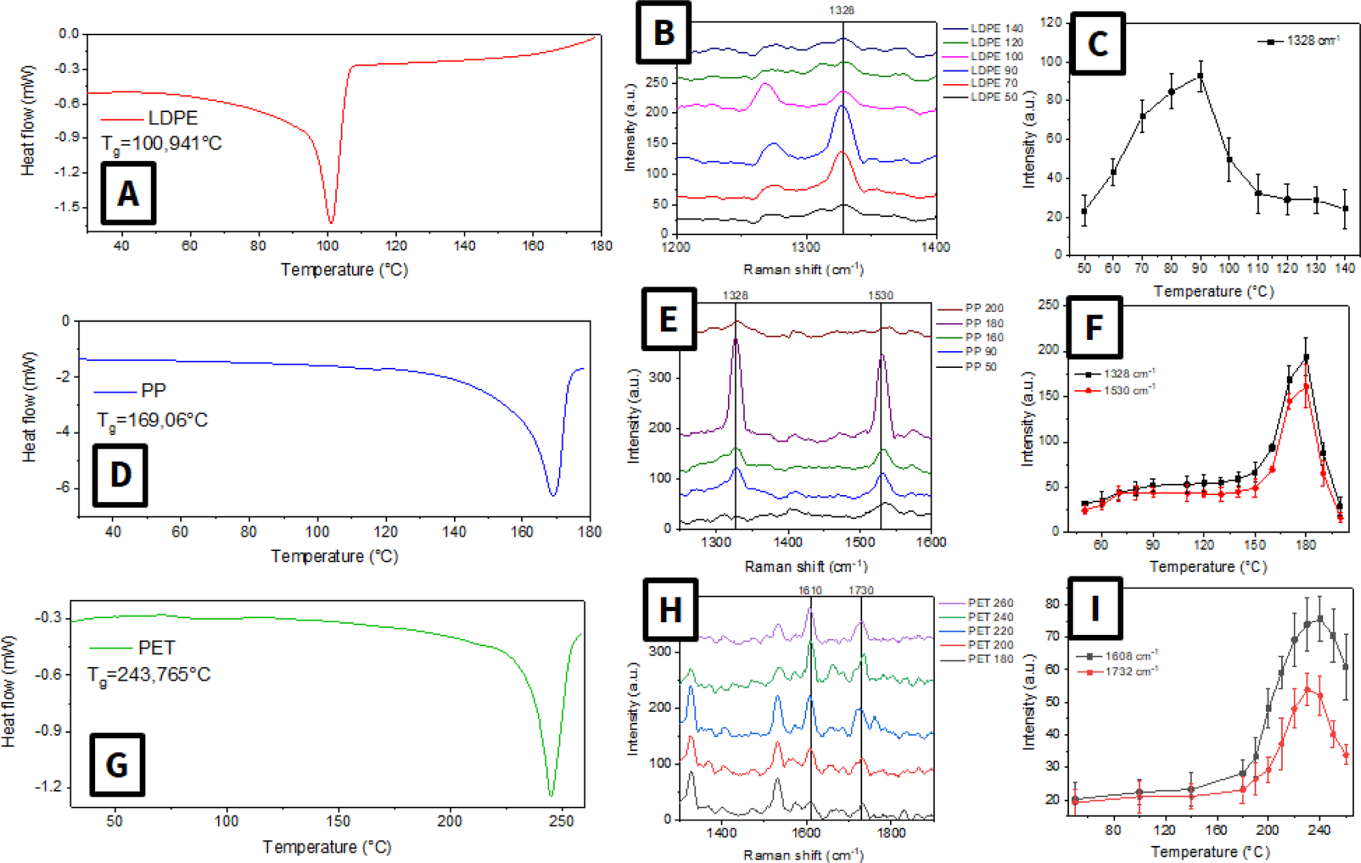
(A, D, G) DSC curves of LDPE, PP, and PET microparticles; (B, E, H) averaged SERS spectra as a function of the annealing temperature of corresponding MPs; (C, F, I) dependence of the characteristic peak intensity of SERS of the polymer (1328 for LDPE, 1328 and 1530 for PP, and 1608 and 1732 cm^–1^ for PET) on the annealing temperature.

The concentration dependencies of averaged SERS spectra (measurements were performed at 30 randomly chosen points, and spectra were subjected to baseline corrections) are presented in Figure 5A for PS microspheres deposited from pure water and in Figure 5B for spectra from PS samples in simulated groundwater containing a mixture of molecules from 3 natural substances: humic acid (HA), tannic acid (TA), and alginic acid (AA). Spectra were measured after annealing at the optimal temperature (120 °C) and evaluated manually. In the first case (Figure 5A), a good SERS response was observed for 10^8^ and 10^7^ particles/L concentrations. The characteristic peaks of PS are also well evident even with a concentration of 10^5^ particles/L, but no characteristic peaks were observed for a 10^4^ particles/L concentration. However, the use of the addition of natural substances (i.e., previous mixing of PS with HA, TA, and AA) results in a significant worsening of the SERS performance. The characteristic PS peaks were screened off by the spectral background (as well as spectral averaging), especially in the case of lower PS microparticle concentrations. In particular, characteristic PS peaks are noticeable only in the case of the highest concentration, 10^8^ particles/L, and are difficult to distinguish for lower concentrations. Indeed, in this case, the manual analysis of spectra can result in operator error and incorrect conclusions about the presence and concentration of PS microparticles. Furthermore, only a part of the SERS spectra contains signals from PS, complicating the identification of PS using averaged spectra or measurements at random points (Figure S9). Moreover, simple spectral averaging results in the almost complete disappearance (especially in the case of simulated real samples with the addition of humic acids) of the characteristic PS signal (Figures S10–S12). For this reason, we utilized an artificial intelligence-based approach for subsequent spectral analysis, with the aim of enhancing the detection limit and maximally simplifying and automating the detection procedure.

**Figure 5:**
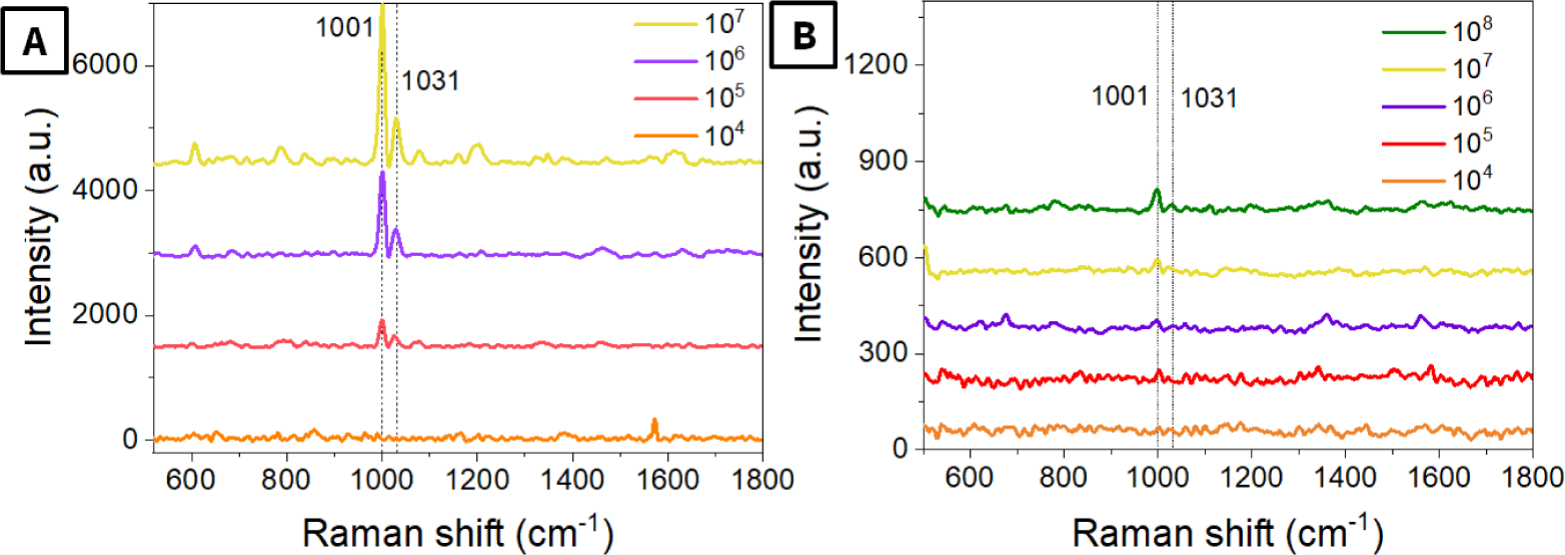
Averaged SERS spectra (conditions: 785 nm, 2 mW, 10 s, 20 average), measured as a function of PS microparticle concentration, deposition was performed from (A) pure water and (B) simulated groundwater accordingly (corrected baseline). Measurements were performed after annealing at 120 °C, at 30 randomly chosen points, and the results are given as an “averaged” spectrum.

At this stage, it should be noted that the utilization of ANN can allow for the analysis of sophisticated spectra and determine the presence/absence of targeted molecules despite peak overlapping and interference. On the other hand, the common ANN makes a decision based on a single spectrum. Thus, the utilization of ANN assumes a more or less homogeneous distribution of the targeted compounds across the SERS surface and the presence of their characteristic spectral features in most of the spectra used. However, such a situation is uncommon in the case of MPs, since they are localized only in a few places across the SERS substrate. Thus, their characteristic spectral features will be present only in a small subset of the spectra collected from the given sample, especially in the case of low MP concentrations. Indeed, our first attempts with the utilization of ANN were far from ideal one (Figures S13 and S14 and a related remark in Supporting Information). We decided to proceed with the transformer architecture, incorporating newly developed KAN layers, which were found to significantly improve training stability, which is a known problem for classical transformers in low-data regimes.^75^ In this case, KANF is able to draw conclusions from multiple spectra, dynamically assigning weights to them depending on their relevance, which is ideal for the MP detection task, because only a fraction of all spectra contain useful information about the PS presence.

The design of the proposed ANN is presented in Figure 6A. Briefly, KANF combines a convolutional feature extractor with a Transformer decoder, which effectively merges information from multiple input spectra, followed by the KAN classification layer, outputting information about MP presence in the sample. The feed-forward counterpart of the attention block is replaced with a KAN layer. The additional motivation for using KAN layers instead of feedforward layers is that they have been found to improve network performance and training stability while having fewer parameters (a detailed comparison between the classical Transformer and KANformer is provided in the Supporting Information). Moreover, KANF allows to estimate the SERS spectral database in the attention-weighted regime, i.e., it mainly takes into account the spectra containing the SERS response of PS, which is significant since only part of the SERS substrate is coated with PS.

**Figure 6:**
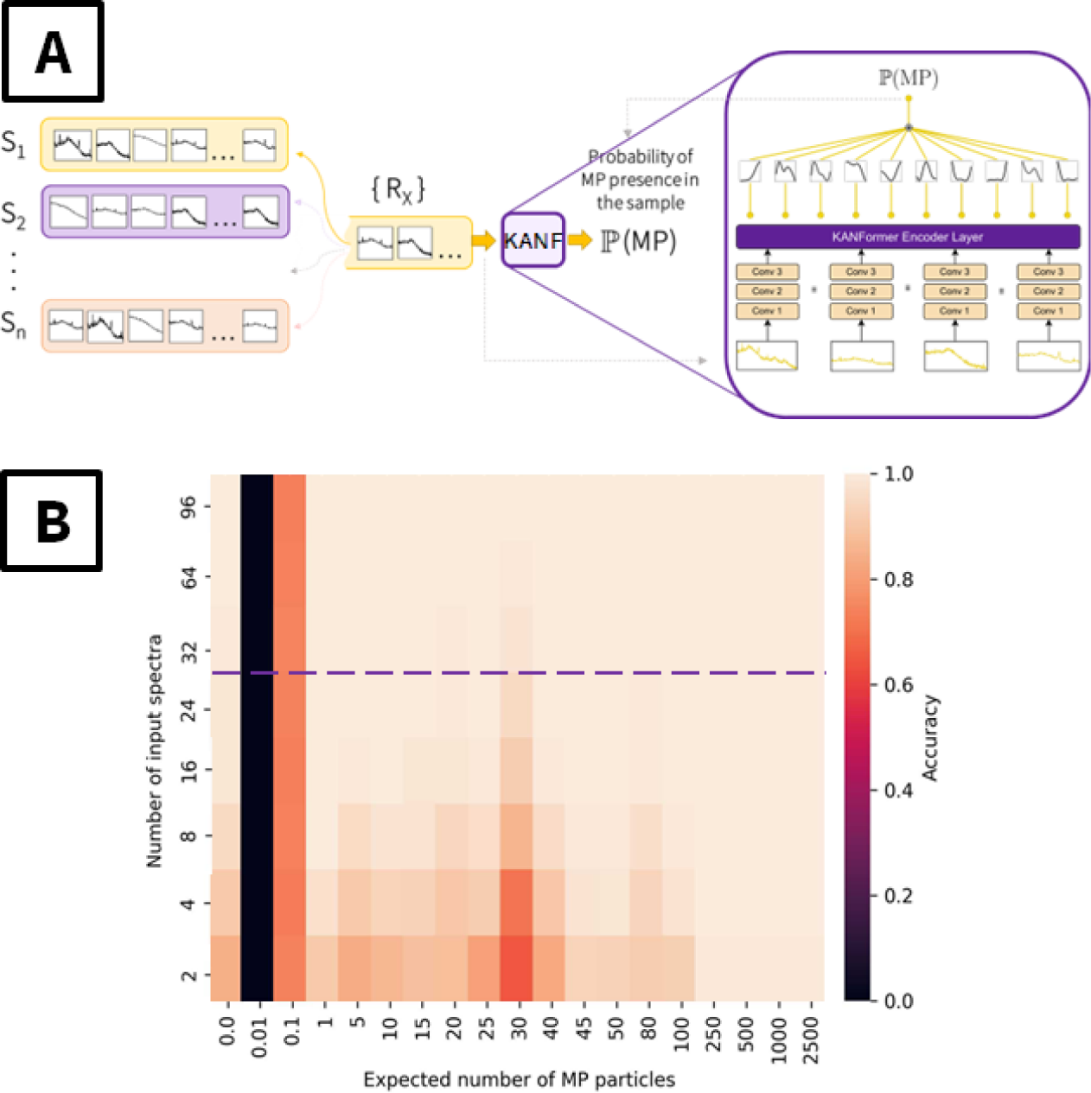
(A) Schematic representation of the KANF design used. During the training phase, a random sample from the training set S_1_...S_*n*_ was selected. A random subset of spectra of the selected sample were used as input to the KANF, which outputs probability of MP presence; (B) results of SERS-KANF approach utilization, in terms of the accuracy dependency on the number of PS microparticles per scanned SERS area (averaged) and the number of SERS spectra collected.

The KANF was trained using the spectra database listed in Table 1 and included the SERS spectra measured from the samples deposited by PS microplastic solutions in pure or simulated groundwater and subjected to annealing. The total number of the samples prepared and used for KANF training and validation was 211. The main results of the SERS-KANF application are presented in Figure 6B, where the determination of the presence of PS by SERS-KANF is plotted as a function of the concentration of particles (concentration of *x* particles per sample) and the number of SERS spectra collected from one sample. As is evident, for a higher PS concentration, the detection accuracy immediately reached a value of 1.0, despite the number of the spectra collected. Apparently, in this case, almost all of the sample surface was covered with the PS material, softened particles penetrated into pores, and the measurement of 1–5 spectra was enough to immediately determine the presence of MPs. With a decrease in PS concentration, the expected number of MPs per scanned area decreased, and a larger number of collected SERS spectra was required for the identification of PS presence. For the expected 50–100 nanoparticles (corresponding to 5 × 10^6^–10^7^ particles/L PS concentration), the collection of 8–10 spectra was required for a correct decision on the presence of MPs. In turn, for a very small PS concentration (5 × 10^5^–10^6^ particles/L), where only 5–50 particles are deposited on the entire sample surface, the SERS-KANF can clearly detect the presence of PS but requires a higher number of spectra (up to 25–30). In this case, even a single PS nanoparticle can be detected on a SERS substrate using the SERS-KANF combination. A further decrease in the PS concentration in the drop-deposited sample results in the deposition (on average) of only one particle on one of 10 or even 100 substrates, and, as could be expected, detection of MPs using SERS-KANF is useless in this case. Thus, a concentration above 10^5^ particles/L (or at least the presence of one PS particle on the SERS scanned area) can be considered the detection limit of the present approach. It should also be noted that when the expected number of particles is 1, the probability of actually having at least one particle on the sample is approximately 0.63. If it happens that the particle is absent in the sample labeled as a positive one, the sample becomes the mislabeled one. Thus, a higher number of samples and spectra are required for a correct decision on PS presence. In the case of 0.1 PS concentration, the Poisson modeling shows that over 90% of the substrates would have no particles at all. Thus, making a correct decision about PS presence with higher accuracy is almost impossible in this case. In the absence of PS microparticles, we reached the correct answer “NO” for measurements of approximately 15–20 spectra in all cases, including the separately prepared samples with different combinations of AA, TA, and HA molecules.

Finally, we performed a range of blinded experiments, where separately prepared samples with or without addition of PS microparticles were measured (30 spectra from each of three substrates for each sample were measured) and subjected to SERS-KANF analysis. Blinded experiments also included a few real samples, where the groundwater samples were randomly collected outdoors and mixed with PS nanoparticles. The composition of the samples was previously unknown to the KANF, and it had to answer the question if the MPs were present or not (the probability of output was regulated at *p* = 0.5, which means that the output of KANF < 0.5 was interpreted as “NO”). The results of the blinded validation approach are summarized in Table 2. We received the correct answer “NO” for all samples without the addition of PS. We also received the correct answer “YES” for samples with concentrations of 10^7^, 10^6^, and 15 × 10^5^ particles/L of PS particles, corresponding to an average deposition of 100, 10, and 15 particles per SERS scanned area. Moreover, similar results were obtained in experiments with real samples (addition of PS to groundwater samples), where successful detection of MPs up to a concentration of 10^6^ particles/L was demonstrated. In turn, the use of prefiltered water showed the absence of microplastics (i.e., the absence of false positive results). However, for 10^5^ PS particle concentration (1 or 0 particles per scanned area, due to some randomness in deposition), the incorrect answer “NO” was also received, which is well correlated with the results presented in Figure 6B.

**Table 2:**
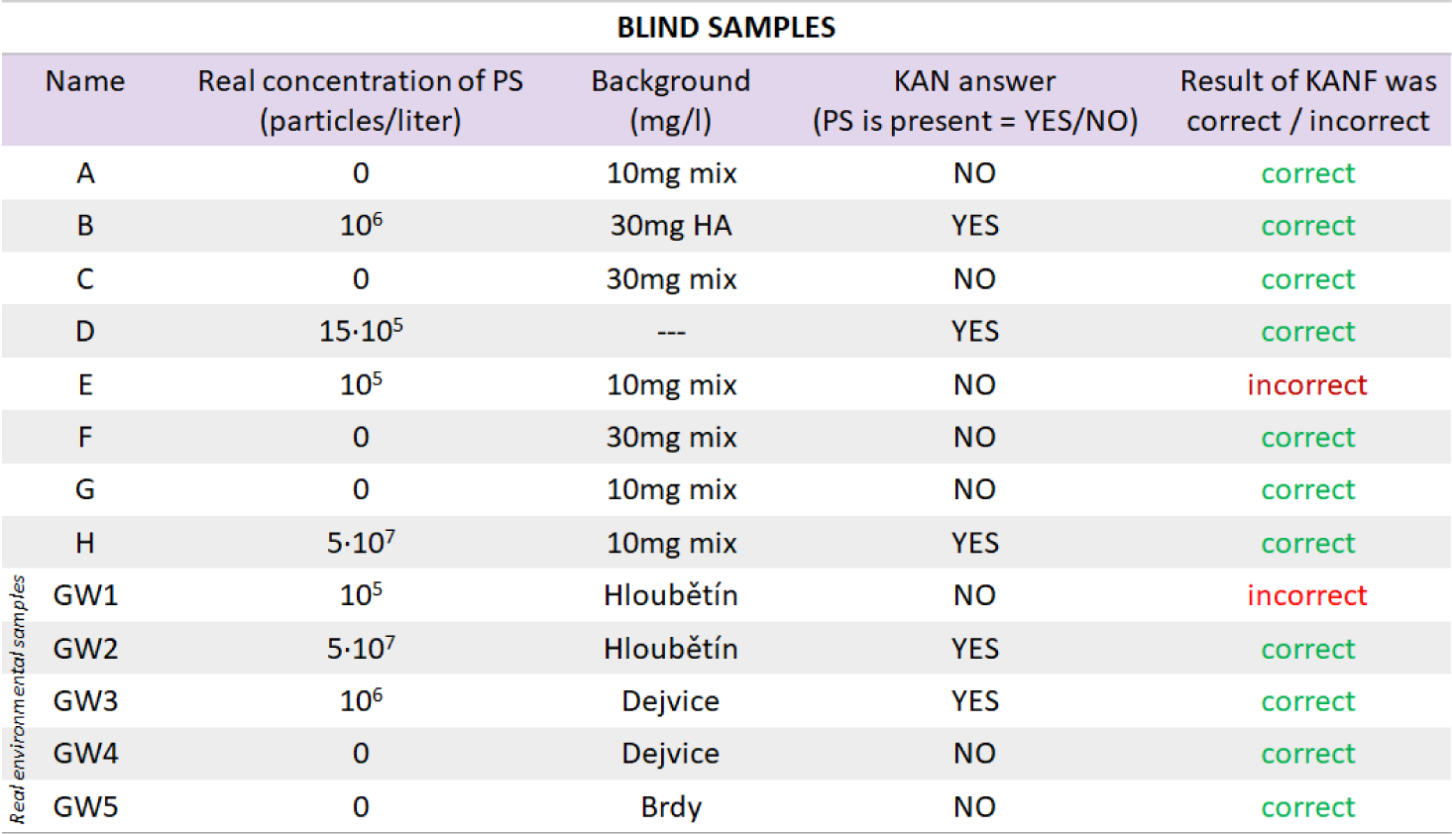
Results of the SERS-KANF Evaluation of Blind Samples with Previously Unknown Composition

We also compared our results with the previously published ones (Table 3).^35–73^ As can be seen, most of the works used model systems that can give good results in the case of laboratory studies but can hardly be used for the analysis of real samples, in which the SERS signal will be obtained from the contaminated surface of the plastic (thus not carrying information about the composition and presence of plastic). Alternatively, several methods of MP pretreatments were proposed. For example, methods such as MP agglomeration, dissolving, or surface treatment using oxidizing agents can be mentioned. However, these approaches are based on the use of sufficiently toxic reagents, with related prolongation of the analysis time and limited specificity (for example, not all plastics dissolve in the same universal solvents). On the other hand, in our research, there is no need for pretreatment of MPs or their dissolution. Moreover, our method is relatively fast and universal, as demonstrated in Figures 4 and S6.

**Table 3:** Comparison of Our Results and Approach with Those Previously Published

Microplastic	SERS substrate	Pretreatment method	Reference
PS (PE not successful)	AuNPs	addition of an aggregating agent	Mikac et al.^35^
PS, PET, PC	AuNPs on the gas–liquid interface	MPs were introduced into the oil phase → the complete evaporation of the oil phase → the MP-AuNPs are measured	Chen et al.^76^
PS	AuNUs (urchin-shaped)	adding NaCl as a coagulant	Lee et al.^34^
PS	AuNSs@Ag@AAO (nanostars)	– – −	Lê et al.^53^
PS (PMMA way less efficient)	Klarite	rinsed in a H_2_O_2_ solution (30%) for 24 h → filtered with a glass fiber filter → rinsed with deionized water → concentrated by heating to 60 °C for 24 h → transferred to Klarite substrates	Xu et al.^33^
PS, PE, PP	AgNPs	– – –	Lv et al.^30^
PS	AgNPs	KI as a coagulant and cleaner to remove surface impurities	Hu et al.^31^
PS	AgNPs	MgSO_4_ as a coagulant, drying at 60 °C	Zhou et al.^77^
PS	AgNW/RC	the analyte solution was vacuum filtered onto hydrogel nanocomposites, then peeled off the filter membrane, and dried (60 °C for 1 h)	Jeon et al.^78^
PS, PE, PMMA, PTFE, nylon, PET	AgF@AgM@C10	– – −	Guselnikova et al.^73^

In summary, in this work, we combine the highly homogeneous SERS substrate with an alternative approach for the introduction of plastics into the plasmonic hot spots. Such an approach allows us to detect a very low concentration of microplastics. However, even in the case of low concentrations, some uncertainty can occur since only a small part of the plasmonic hot spots is occupied by the polymer material. To overcome this drawback (and to avoid additional steps of sample purification), we propose the utilization of advanced KANF design. Unlike classical ANN, which analyzes a whole array of spectra, KANF analyzes a random sample of spectra with a variable composition. We also estimated the time required for SERS-KANF analysis (after the preliminary KANF training and validation). Drop deposition and heating/cooling of the samples took 10 min. The subsequent collection of 30 spectra (the number of spectra was determined from Figure 6B) takes approximately 25 min. The final evaluation by KANF takes a very short time: less than 1 s. The proposed SERS-KANF combination takes approximately 35–40 min and can determine even a single PS particle on a SERS-active surface, without the need for any preliminary sample treatment, separation, or purification.

## Conclusion

In this work, we propose the SERS-ANN approach (in particular, SERS-KANF) for the detection of microplastic presence. The detection was performed with the utilization of simple PS nanoparticles dispersed in pure water and PS mixed with simulated organic molecules commonly present in groundwater. Unlike the previously published approaches, the PS efficiently entered “inside” the plasmon-active pores created on the porous Si surface and was subjected to strong plasmon triggering. This was achieved by heating the PS sample at the optimized temperature. The heating immediately resulted in a significant increase in the SERS response from the PS microparticles. The design of KANF was optimized to meet the main detection goal, taking into account that only part of the substrate is covered by deposited PS, and thus only a limited number of SERS spectra can contain characteristic PS signals (especially in the case of lower number of PS particles). After collecting the SERS database and performing appropriate KANF training and validation, we demonstrated that the detection of even a single PS nanoparticle on a SERS-active substrate is possible. Moreover, the successful determination of single (or few) PS nanoparticles was demonstrated independently in the presence of background molecules from simulated groundwater. We also demonstrated that detection accuracy increased significantly with an increase of the SERS spectra number and found the optimal number of spectra that should be collected for the detection of a single PS particle. These results were further validated with utilization of blinded, independently prepared samples with a composition previously unknown to KANF. Finally, it should be noted that SERS-KANF-based PS detection is easy and fast, and the total time required for the analysis is only 35 min.

## Supplementary Material



## Data Availability

The data that support the findings of this study are openly available on Zenodo at https://zenodo.org/records/14996110.

## Notes

Experimental section (materials used, sample characterization details); SEM images of the porous silicon surface and PS microparticles; calculation of the SERS enhancement factor; SERS and Raman spectra of PS and background bulk groundwater substances; SERS map over the sample with PS; dependence of characteristic PS peak intensity on the annealing time and corresponding weight loss; averaged unprocessed Raman spectra of samples containing PS reference and HA, TA, AA background substances; different concentrations of PS and different concentrations of PS with different background substances; comparison of validation loss and accuracy evolution during training for classical Transformer and Transformer with KAN layers (KANF); description of the difference between the traditional Transformer and KANformer (PDF)
