# Supplementary material for: Detection of Polystyrene Microplastics up to the Single Nanoparticle Limit Using SERS and Advanced ANN Design (KANformer)
Source: ACS Sens. 2025 Jun 25;10(7):4983–95. doi: 10.1021/acssensors.5c00846 (PMC12305665; doi:10.1021/acssensors.5c00846)
Supplement: Supplementary file 1 [file se5c00846_si_001.pdf]

SUPPORTING INFORMATION

for:

Detection of polystyrene microplastics up to the single nanoparticle limit using SERS and advanced ANN design (KANformer)

Karolina Kukralova, Andrii Trelin, Elena Miliutina, Vasilii Burtsev, Vaclav Svorcik, Oleksiy Lyutakov*

Department of Solid State Engineering, University of Chemistry and Technology, 16628 Prague, Czech Republic.

---

*Corresponding author: oleksiy.lyutakov@vscht.cz

Experimental section

Materials

Hydrofluoric acid (HF, 48.0 %); Crystal Violet (tris(p-dimethylaminophenyl)methyl chloride); Dimethylformamide (DMF, ≥ 99.8 %); humic acid sodium salt (HA), alginic acid sodium salt (AA), tannic acid (TA) and ethanol were purchased from Sigma Aldrich. The Si wafers used (p-type, boron-doped, (100) crystallographic orientation, resistivity 0.5–1 Ω × cm) were produced by Siegert wafer. Polystyrene was used in the form of microsperes (PS, latex, 2.5 wt. % dispersion in water, diameter 0,5 µm, AlfaAesar).

Samples characterization.

The Differential scanning calorimetry spectra were measured with DSC 3+ (Mettler Toledo, Switzerland), in the temperature range 25–180° C in 3 cycles (heating and cooling) at standard pressure and air atmosphere. The UV-Vis absorption spectra of the substrates were obtained using an HR2000 spectrometer (Ocean Optics) in the 400 – 1000 nm wavelength range with AvaLight-DHS light source (Avantes). Scanning electron microscopy (SEM) and energy-dispersive X-ray spectroscopy (EDX) (LYRA3 GMU, Tescan, CZ) were collected for 20 min at an operating voltage of 10 kV and a beam current of 600 pA. Surface polarity was estimated using liquid contact angle measurement (Krüss DSA 100 device). The volume of liquids drop (water and glycerol) was 4 µL.

All the SERS spectra were collected using a 785nm laser wavelength at DXR3 Raman Microscope (Thermo Scientific) (conditions: 5 s, 10 average, 2 mW laser intensity). The baseline correction was performed in OMNIC.

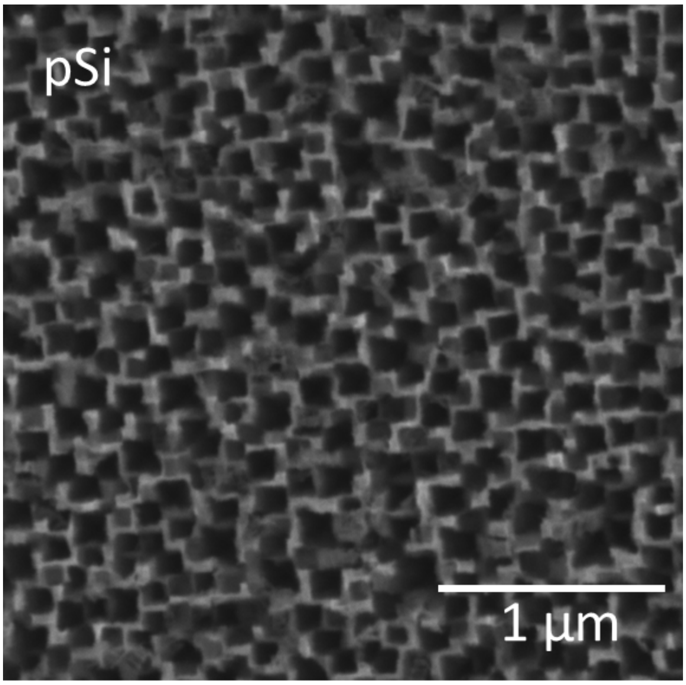

Figure S1. SEM image of porous silicon surface.

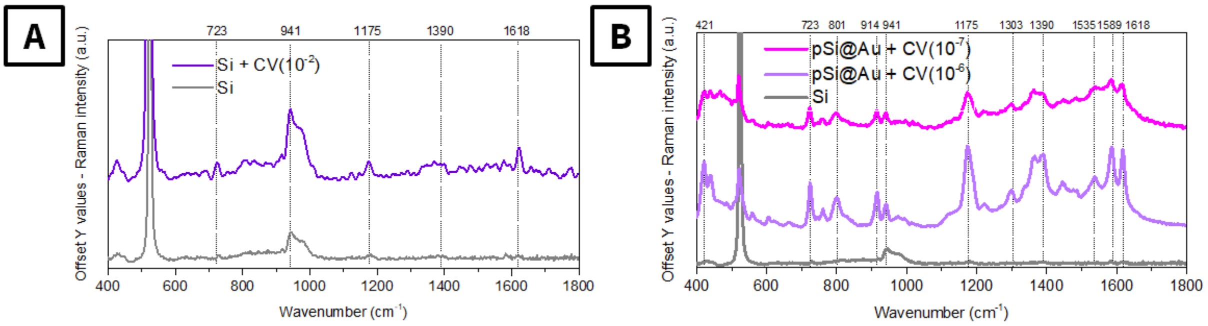

C

$$EF = \frac{\frac{I_{SERS}}{c_{SERS}}}{\frac{I_{RS}}{c_{RS}}} \qquad EF_{pSi@Au} \approx 10^6$$

Figure S2. SERS spectra measure on: (A) – porous Si substrate (CV concentration 10^{-2} mol/L); (B) – plasmon active pSi@Au (CV concentration 10^{-6} and 10^{-7} mol/L), deposition conditions: spin coating, 1500 rpm speed, 1000 rpm/s acceleration, 60 s. spin-coating time, 50 µL drop volume; (C) – calculation of SERS EF (c is the concentration of CV used on pSi@Au (c_{SERS}) or non-SERS substrate (c_{RS}) and I is the intensity of a specific peak of CV corresponding to pSi@Au (I_{SERS}) on non-SERS substrate (I_{RS}))[S2].

Figure S2 – remark

Calculation of the SERS enhancement factor

For the calculation of SERS EF, the different concentration of crystal violet were spin-coated on pSi (Figure S2A) and pSi@Au (Figure S2B) substrates. Subsequently, Raman or SERS spectra were

collected, and the intensity of the characteristic peak (located at 1175 cm^{-1}) was estimated. The SERS EF was calculated using the equation presented in Figure S2C. The value of EF was found to be 10^6.

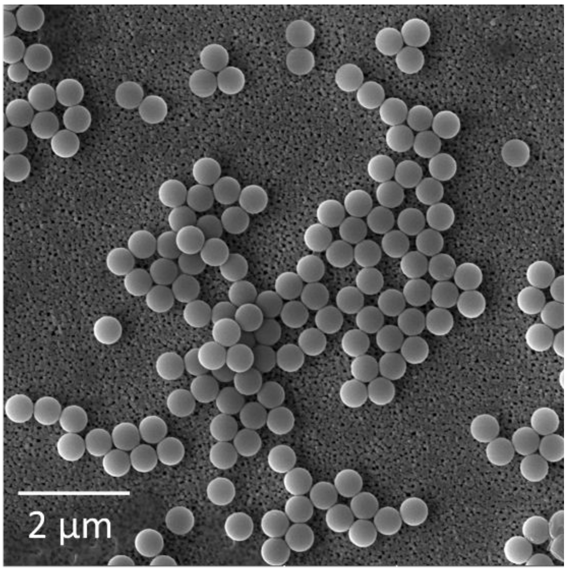

Figure S3. SEM image of PS microparticles deposited on a flat surface.

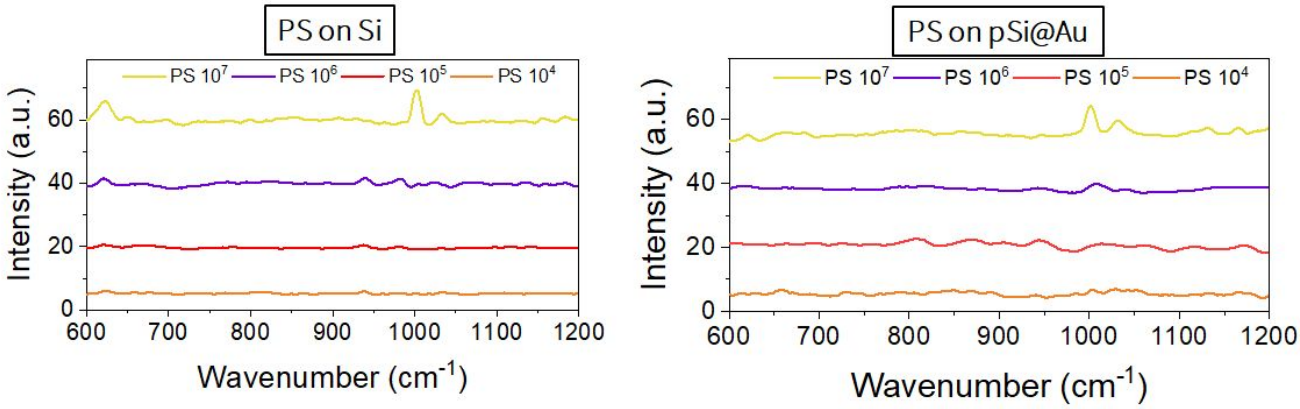

Figure S4. SERS spectra of PS on a pSi/Au substrate without thermal annealing (high and low concentrations) vs. Raman spectrum of PS deposited on a flat Si substrate.

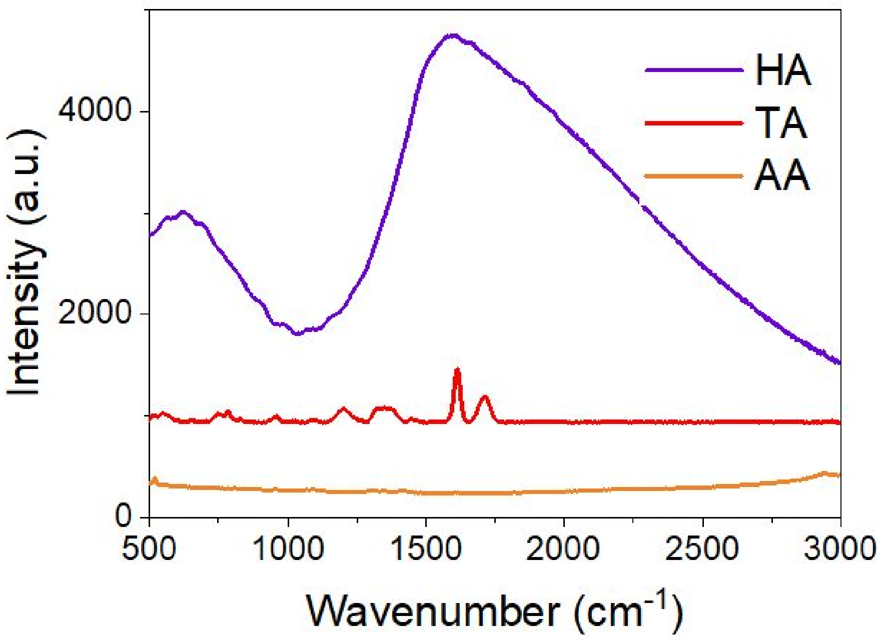

Figure S5. SERS spectra of background bulk groundwater substances (humic acid – HA, tannic acid – TA, alginic acid – AA, c=1500 mg/L).

Figure S5 – remark

The presence of 'background' compounds in the simulated groundwater water sample produces additional signal and significantly complicates PS detection. In particular, during measurement, HA produces a significant fluorescence band, TA acids presence results in additional SERS bands, while AA molecules do not create the significant spectral background, but can occupy the plasmonic hot spots, restricting the triggering of PS material by plasmonic evanescent wave.

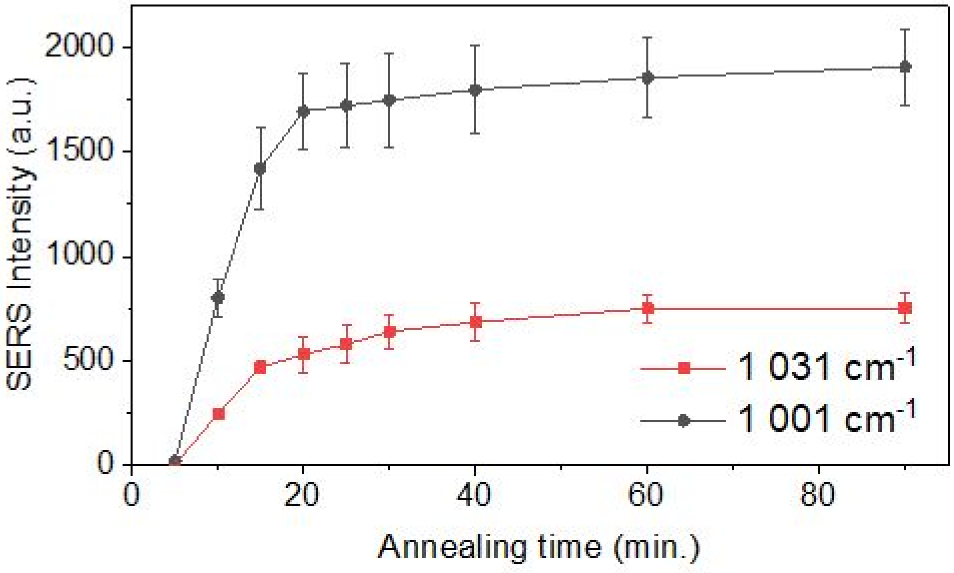

Figure S6. Dependence of characteristic PS peaks intensity on the annealing time (120° C).

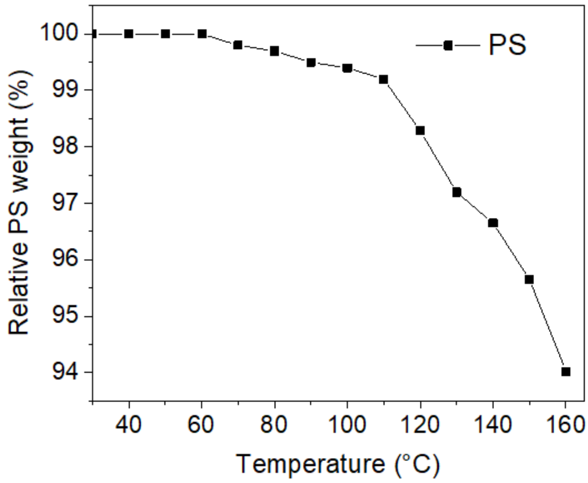

Figure S7. Relative weight (weight loss) of PS microparticles as a function of annealing temperature.

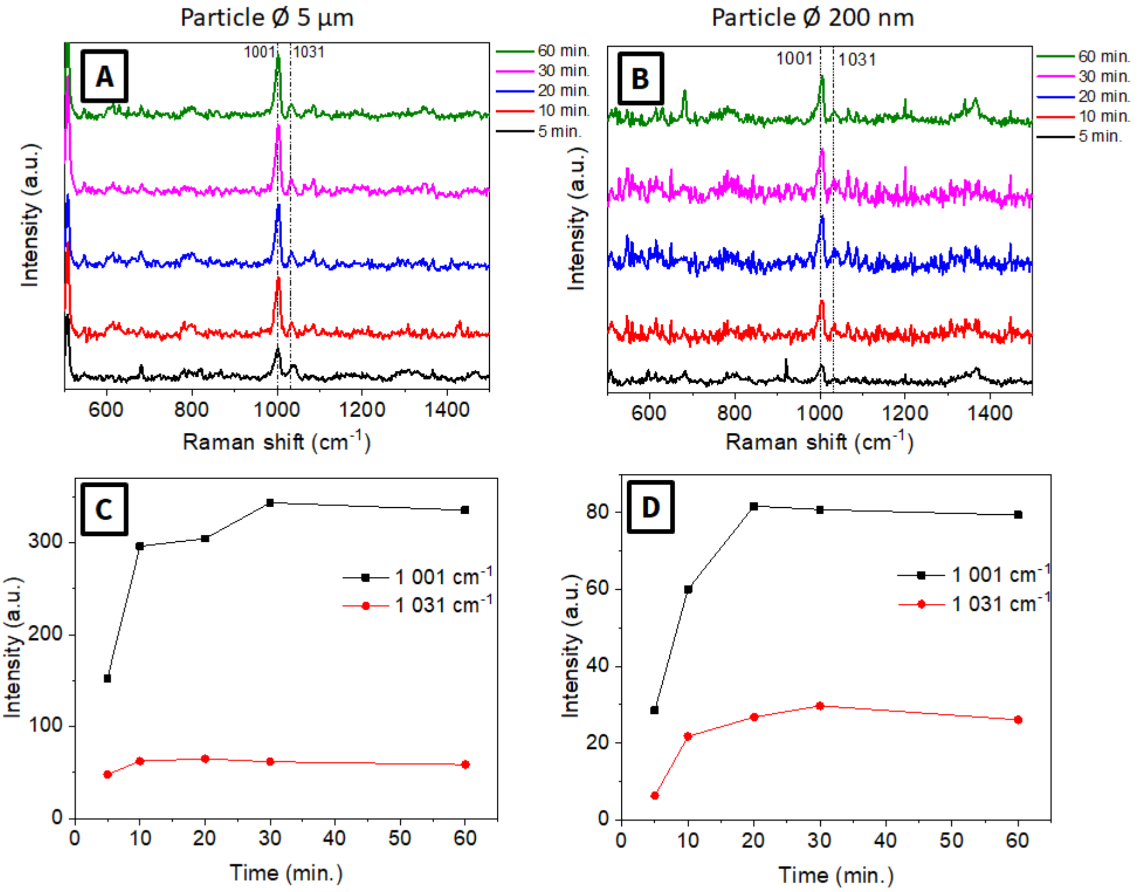

Figure S8. SERS spectra obtained from samples with different PS particle size under annealing at 120 °C (A) 5 µm and (B) 200 nm particles size; (C, D) – corresponded increase of characteristic SERS peaks intensity.

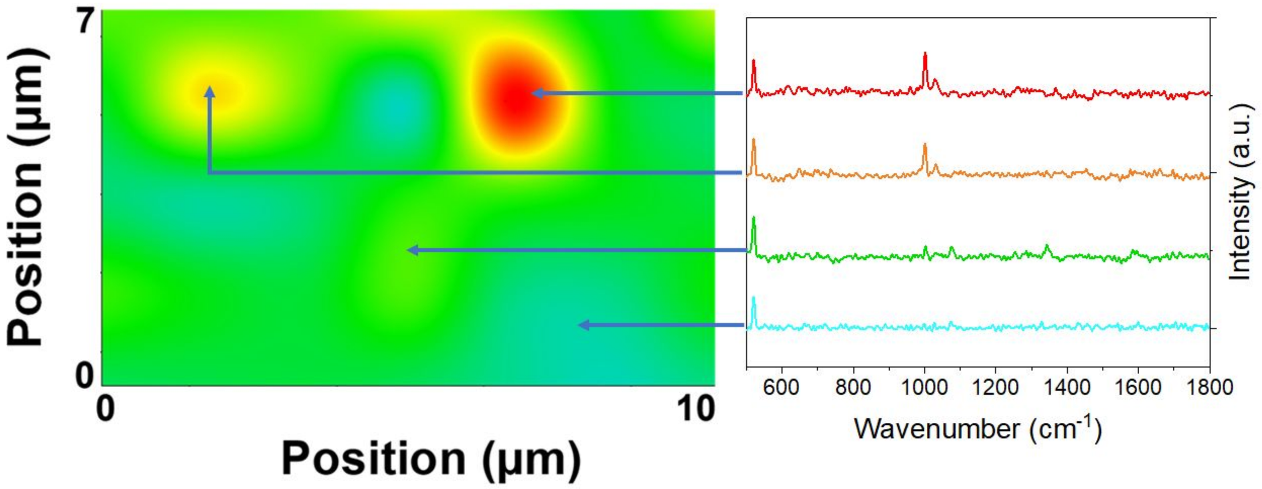

Figure S9. Characteristic map according to the distribution of PS peak (1 001 cm^{-1}) (the concentration of PS in the sample is 10^5 particles/L, selected area) and selected spectra (baseline corrected).

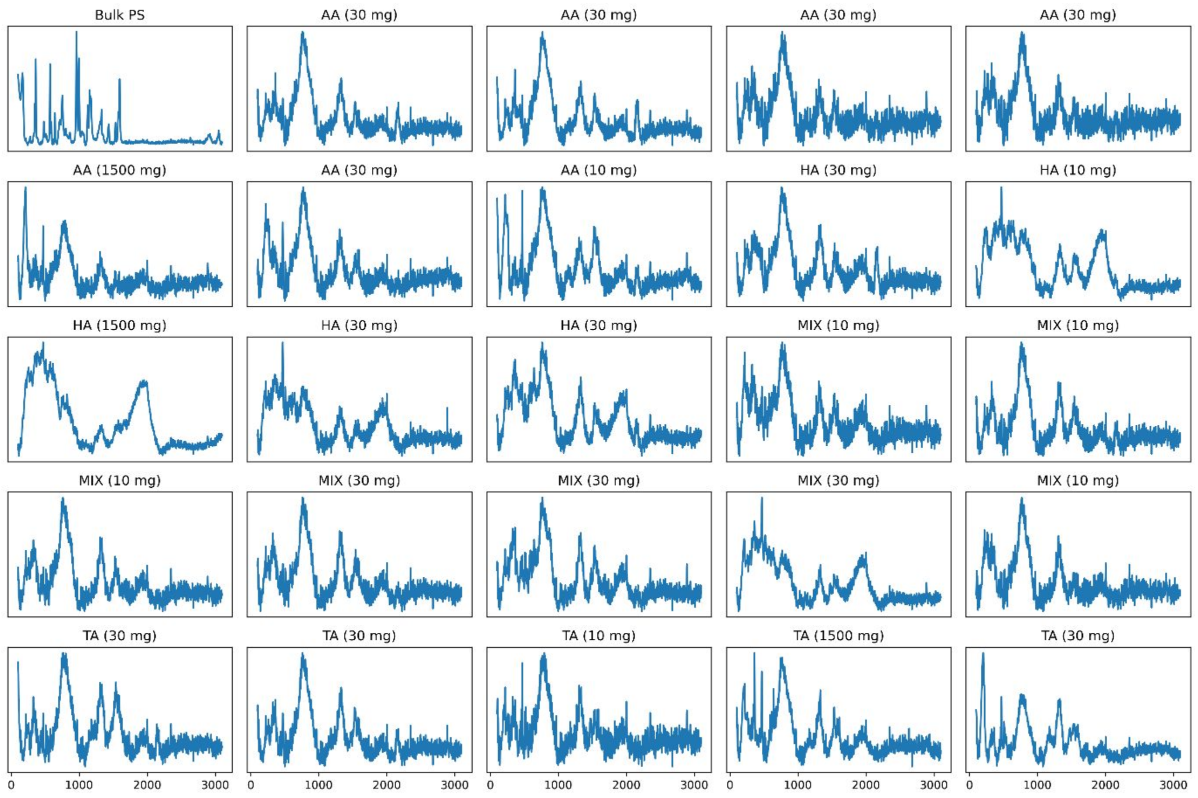

Figure S10. Averaged unprocessed Raman spectra of samples containing PS reference and HA, TA, AA background substances.

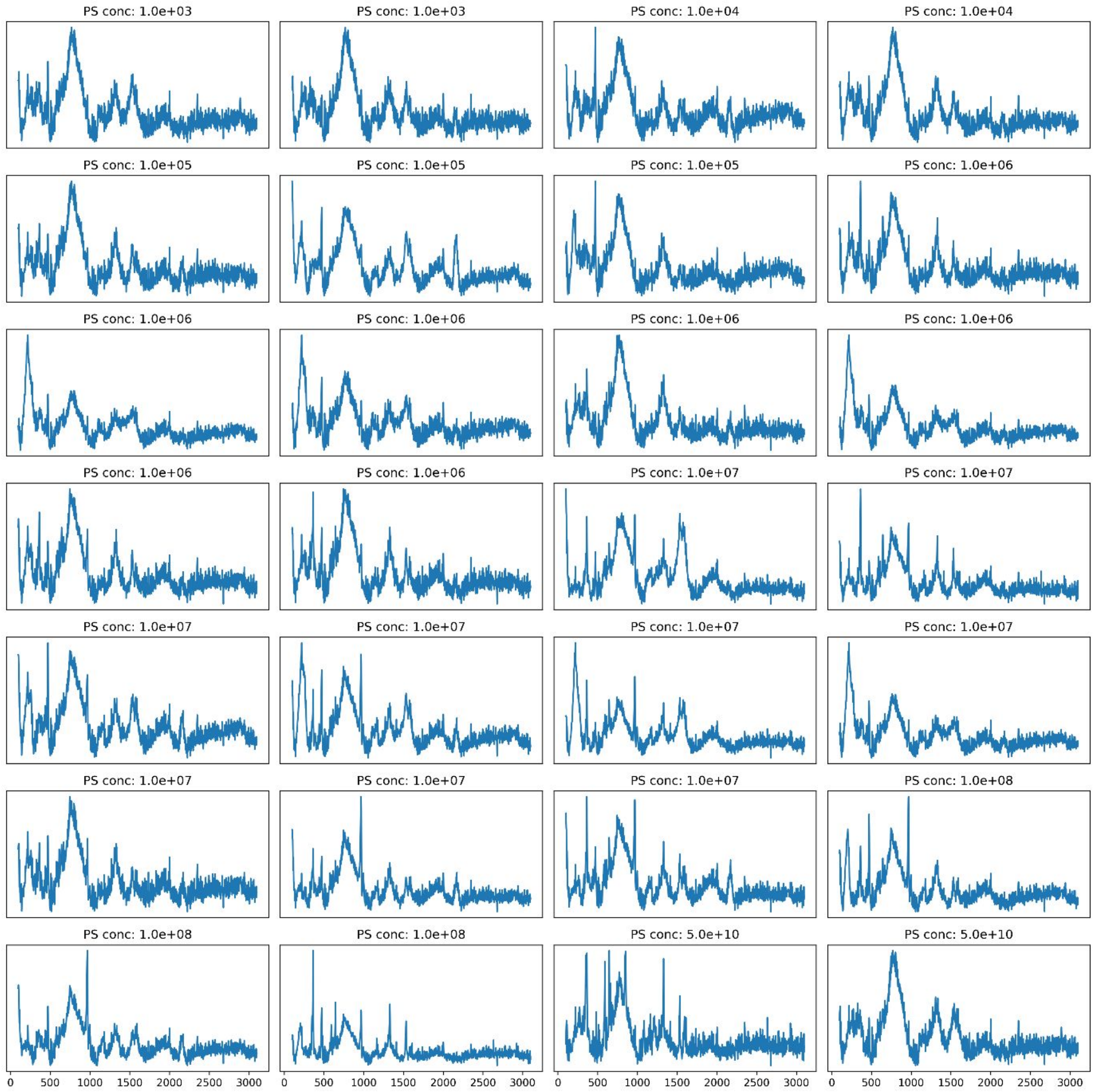

Figure S11. Averaged unprocessed Raman spectra of samples containing different concentrations of PS.

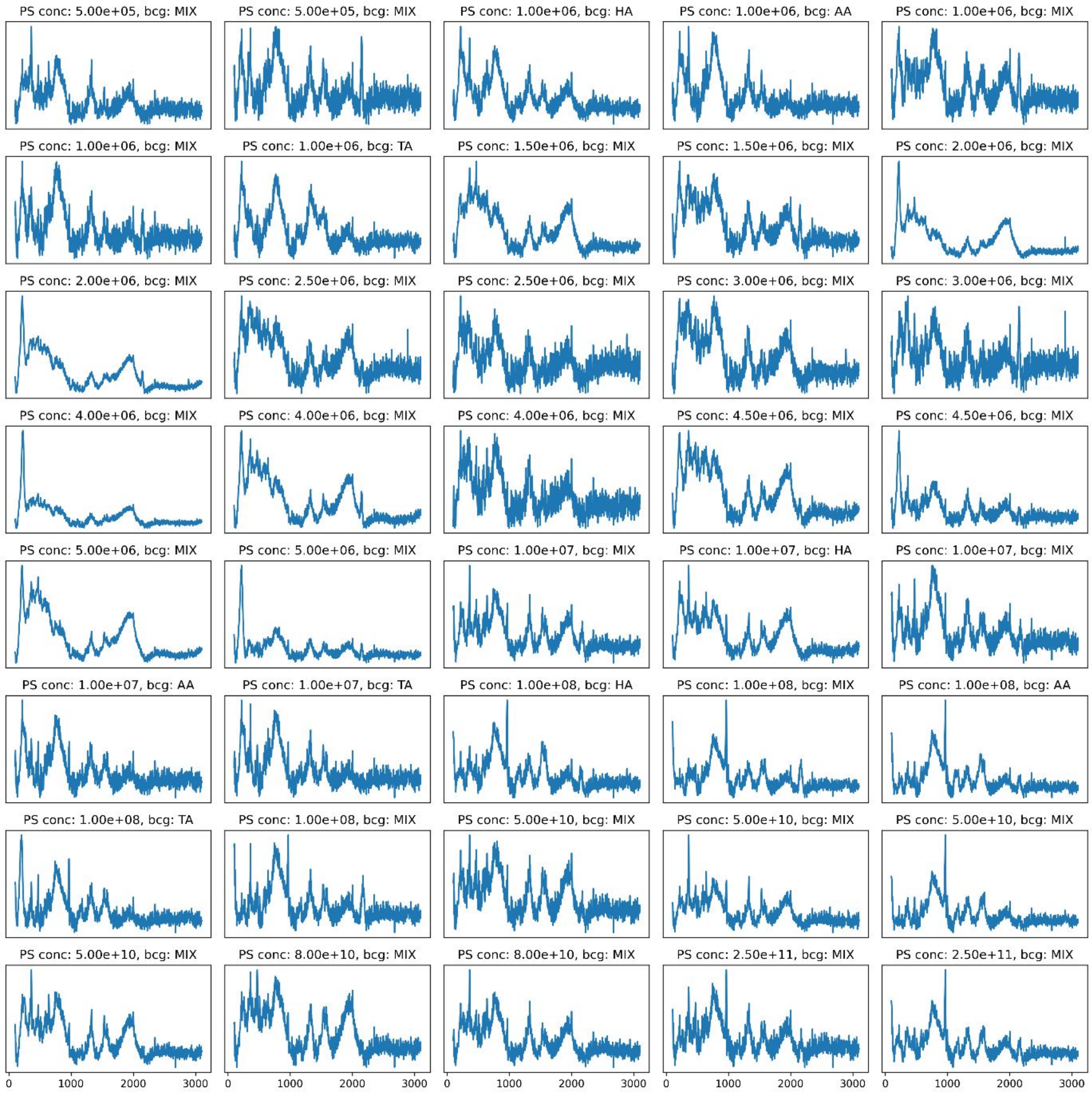

Figure S12. Averaged unprocessed Raman spectra of samples containing different concentrations of PS as well as different background substances.

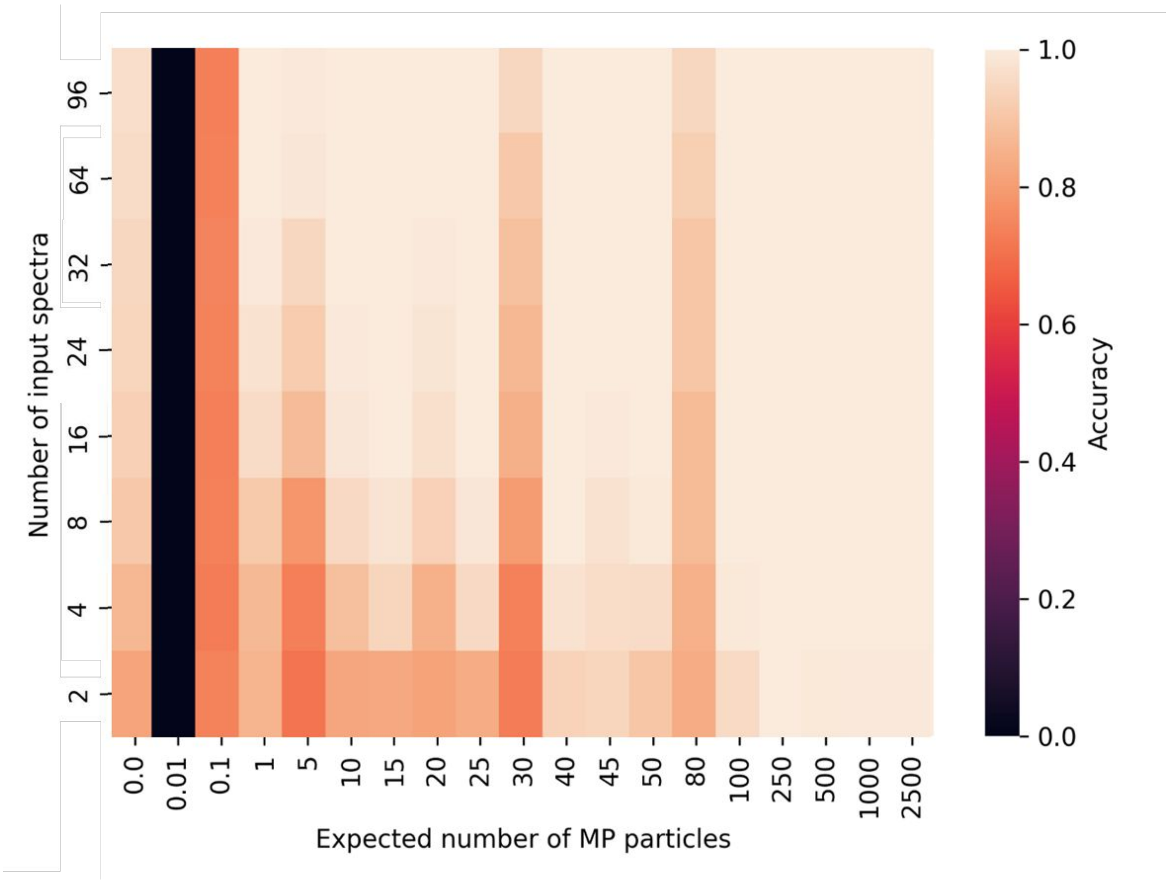

Figure S13. Results obtained using a traditional transformer with feed-forward layers.

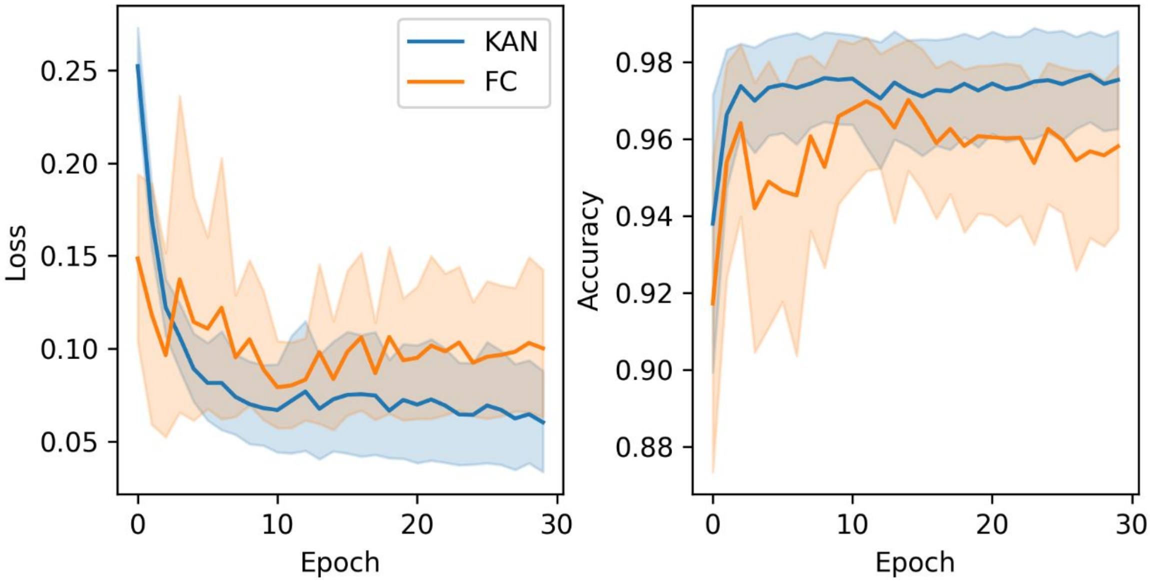

Figure S14. Comparison of validation loss and accuracy evolution during training for classical transformer (with fully-connected layers, FC) and transformer with KAN layers (KANF). Much smoother blue curves demonstrate higher training stability. Shaded band shows a 90 % confidence interval.

Figures S13 and S14 – remark

Traditional transformer vs KANformer

To demonstrate improvement of training stability and final accuracy of transformer + KAN architecture compared to classical transformer, an additional experiment was conducted. Specifically, the training process of both networks was repeated 10 times using different training-validation splits. Results, reached with utilization of common transformer are presented in Figure S13. Comparison with results of KANF utilisation (Figure 6B vs. Figure S14) indicates that KANF can ensure a significantly better analysis reliability and required a lower amount of spectra collection for a correct decision. In addition, history of loss and accuracy evolution during training process were saved and plotted in the Figure S14, clearly demonstrating both the higher stability of the training process of KANF version of the network as well as better metrics achieved (higher accuracy and lower loss).

References

[S1] Kukralova, K.; Miliutina, E.; Guselnikova, O.; Burtsev, V.; Hrbek, T.; Svorcik, V.; Lyutakov, O. Dual-mode electrochemical and SERS detection of PFAS using functional porous substrate. Chemosphere **2024**, 364, 143149. DOI: https://doi.org/10.1016/j.chemosphere.2024.143149.

[S2] Le Ru, E. C.; Blackie, E.; Meyer, M.; Etchegoin, P. G. Surface Enhanced Raman Scattering Enhancement Factors: A Comprehensive Study. The Journal of Physical Chemistry C **2007**, 111 (37), 13794-13803. DOI: https://doi.org/10.1021/jp0687908.
